# PW01-001 – Pyrin-PSTPIP1 relation during cell migration

**DOI:** 10.1186/1546-0096-11-S1-A54

**Published:** 2013-11-08

**Authors:** ZY Akkaya, B Balci-Peynircioglu, A Cetinkaya, N Purali, E Yilmaz

**Affiliations:** 1 Medical Biology, Hacettepe University, Ankara, Turkey; 2 Medical Genetics, Hacettepe University, Ankara, Turkey; 3 Biophysics, Hacettepe University, Ankara, Turkey

## Introduction

MEFV (MEditerranien FeVer) gene mutations cause Familial Mediterranean Fever (FMF). This gene encodes a protein termed as Pyrin, which appears to play an important role in the inflammatory pathways. It is far characterized that Pyrin, which is expressed in neutrophils, interacts with PSTPIP1 and actin proteins. In previous studies PSTPIP1 has been shown to interact with cell migration proteins and actin polymerization is a main force driving neutrophil migration. Therefore, we hypothesized that Pyrin can play role in cell migration through the interaction with actin and PSTPIP1.

## Objectives

In this study, Pyrin-PSTPIP1 interaction was analyzed during cell migration. Our aim was to investigate whether these two proteins co-localize at the leading edge of the cell, where actin polymerization occurs.

## Methods

A cell migration assay was generated using HL-60 cells. First of all, HL-60 cells were differentiated in to neutrophil like cells by using appropriate concentration of DMSO (DiMethyl SulfOxide). To test the efficiency of differentiation, neutrophil cell surface receptor FPR1 (Formyl Peptide Reseptor 1) gene expression levels were measured. Secondly, after differentiation, cells were stimulated for migration using fMLP (N-formyl-Met-Leu-Phe), a well-known chemoattractant of neutrophils. The suitable fMLP concentration was determined by actin immunofluorescence staining. Neutrophil migration was demonstrated by using Live-cell imaging analysis. Lastly, un-differentiated, differentiated and differentiated-stimulated cells were co-stained for Pyrin-Actin, PSTPIP1-Actin and Pyrin-PSTPIP1 in order to test if the proteins localize together at the leading edge of the cell. Slides were analyzed by drawing profile and correlation curves with the help of confocal microscopy.

## Results

The suitable concentration of DMSO for the experiment was 1,28% DMSO. In this concentration, FPR1 gene expression showed 165,89 fold increase (p<0,0021). After differentiation, cells showed 90% actin polymerization following 2 hours incubation with 150 nM fMLP. In stimulated cells Pyrin-Polymerized actin, PSTPIP1-Polymerized actin and Pyrin-PSTPIP1 are found to be co-localized.

## Conclusion

In differentiated and differentiated-stimulated cells, Pyrin was localized with actin and PSTPIP1 at the leading edge of the migrating cell. Also an interaction between PSTPIP1 and polymerized actin was shown. So far, PSTPIP1 was shown to localize rear of the cell, mostly in uropods. For the first time, PSTPIP1 was found to interact with dynamic actin and Pyrin at the site of polarization. Further studies on the effect of colchicine on this interaction during cell migration are under way. These data may contribute to understand the exact mechanism of cell migration through Pyrin-PSTPIP1 interaction.

## Disclosure of interest

None declared

